# Dysregulation of Bmi1 promotes malignant transformation of hepatic progenitor cells

**DOI:** 10.1038/oncsis.2016.6

**Published:** 2016-02-29

**Authors:** R Zhang, W R Wu, X D Shi, L B Xu, M S Zhu, H Zeng, C Liu

**Affiliations:** 1 Guangdong Provincial Key Laboratory of Malignant Tumor Epigenetics and Gene Regulation and Department of Hepato-Pancreato-Biliary Surgery, Sun Yat-sen Memorial Hospital, Sun Yat-sen University, Guangzhou, China; 2 Department of Pathology, Sun Yat-sen Memorial Hospital, Sun Yat-sen University, Guangzhou, China

## Abstract

Adult hepatic progenitor cells (HPCs) are involved in a wide range of human liver diseases, including hepatocellular carcinoma (HCC). Bmi1 has been reported to have vital roles in stem cell self-renewal and carcinogenesis. We have previously demonstrated that Bmi1 is upregulated in HCC with bile duct tumor thrombi, a subtype of HCC characterized by profuse expression of hepatic stem cell markers. However, the function of Bmi1 in HPCs has not yet been well elucidated. The current study was designed to investigate the effects of Bmi1 on the biological properties of rat HPCs. To accomplish this, Bmi1 was silenced or enhanced in two HPC cell lines (WB-F344 and OC3) by, respectively, using either small interfering RNA against Bmi1 or a forced Bmi1 expression retroviral vector. The biological functions of Bmi1 in HPCs were investigated through cell proliferation assays, colony-formation assays, cell cycle analysis and invasion assays, as well as through xenograft-formation assays. In this study, genetic depletion of Bmi1 repressed cell proliferation, colony formation and invasion in both assessed HPC cell lines relative to controls. Conversely, forced expression of Bmi1 in two HPCs cell lines promoted cell proliferation, colony formation and invasion *in vitro*. Aldehyde dehydrogenase (ALDH) assay revealed a significant increase in the number of ALDH-positive cells following the forced expression of Bmi1 in HPCs. Most importantly, transplantation of forced Bmi1 expression HPCs into nude mice resulted in the formation of tumors with histological features of poorly differentiated HCC. Taken together, our findings indicate that forced expression of Bmi1 promotes the malignant transformation of HPCs, suggesting Bmi1 might be a potential molecular target for the treatment of HCC.

## Introduction

Hepatocellular carcinoma (HCC) is the most common type of primary liver cancer and is the third most frequent cause of cancer-related death worldwide.^1^ Stem cells are a minor cell population that possesses the capability for extensive self-renewal, differentiation and the repair of damaged tissues.^2^ With regard to HCC or intrahepatic cholangiocarcinoma, the carcinogenesis process has been suggested to begin with 'oval cells', which have bipotent differentiation capability and can transform into either hepatocytes or cholangiocytes. This process is known as 'stem cell hit theory', a hypothesis that stem/progenitor cells in normal tissue are a source of cancer.^3^ In clinical pathology, combined HCC and cholangiocarcinoma, which exhibits intermediate properties between liver and biliary tract cancer, is known to exist. Thus, in the liver, carcinogenesis involving stem/progenitor cells as a source of cancer development is thought to be possible.^4, 5^ Based on gain-of-function and loss-of-function analyses, Chiba *et al.*^6^ noted that primary liver cancer could originate from hepatic stem cells isolated from embryonic day 13.5 C57BL/6 mouse fetal livers. It is well known that HCC mainly occurs in adulthood, but it remains unclear whether HCC can also originate from the malignant transformation of adult hepatic progenitor cells (HPCs).

The oncogene B-cell-specific Moloney murine leukemia virus integration site 1 (Bmi1) is an essential co-factor for polycomb-repressive complex 1, which is involved in several cellular processes, including cell cycle regulation, apoptosis and maintenance of stem cells, through conferring a self-renewal capacity.^7^ Bmi1 was first identified to cooperate with the oncoprotein c-Myc to induce B- or T-cell leukemia.^8^ Since this discovery, aberrant overexpression of Bmi1 has been detected in several human cancers, including prostate cancer, colorectal carcinoma, HCC, non-small cell lung cancer, breast carcinoma and glioblastoma.^9, 10, 11, 12, 13, 14, 15^ Increased Bmi1 expression is frequently been observed in HCC and has been reported to sustain the tumor-initiating ability.^16, 17^ Accordingly, we found that Bmi1 was highly expressed in HCC with bile duct tumor thrombi,^18^ a subtype of HCC with relatively more profuse expression of stem cell markers.^19^ Moreover, we previously demonstrated that Bmi1 knockdown not only decreased proliferation and invasion of HCC cell lines but also significantly increased chemosensitivity.^11^

Recent studies have revealed that Bmi1 has a role in regulating the self-renewal of both normal and cancer stem cells (CSCs). For example, Bmi1 was found to be necessary for the self-renewal of normal hematopoietic stem cells as well as leukemic stem and progenitor cells.^20, 21, 22^Bmi1 has also been shown to regulate the self-renewal and proliferation of CSCs from other tumor types, such as prostate cancer and pancreatic cancer.^9, 23^ Chiba *et al.*^16^ reported that knockdown of Bmi1 markedly decreased side population fractions in HCC cell lines. These side populationcells are believed to exhibit CSCs-like properties.^24^ These findings indicate the important role of Bmi1 in the maintenance of stemness and its possible contribution to the malignant transformation of stem cells. However, the exact role of Bmi1 in driving HPCs carcinogenesis remains unclear. In the current study, we aimed to investigate the biological function of Bmi1 in the malignant transformation of adult rat HPCs (WB-F344 and OC3) and its underlying mechanisms.

## Results

### Transfection of Bmi1-siRNA suppresses the proliferation, colony formation, invasion and cell cycle progression of HPCs *in vitro*

**Figure 1:**
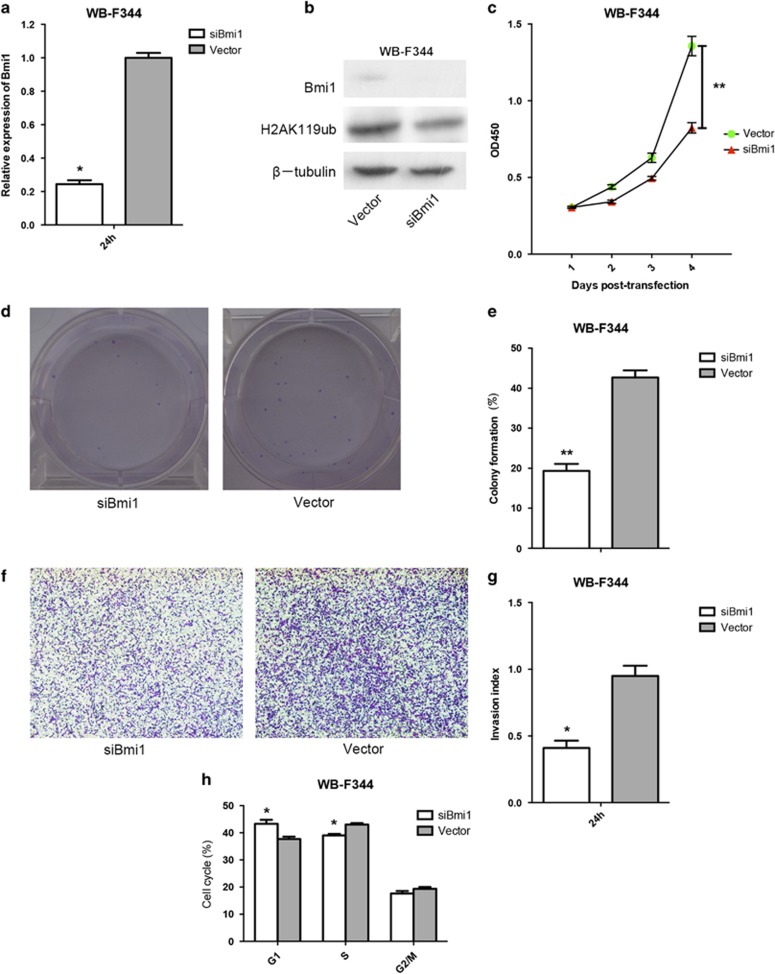
Transfection of Bmi1-siRNA suppresses the proliferation, colony formation, invasion and cell cycle progression of WB-F344 cells *in vitro*. The qRT–PCR analysis revealed that the mRNA expression levels of Bmi1 in Bmi1-siRNA WB-F344 cells were significantly downregulated (**a**). Similar results were obtained through western blot analysis (**b**). The expression of H2AK119 ub was downregulated in WB-F344 cells by Bmi1-siRNA (**b**). Downregulation of Bmi1 expression significantly inhibited the proliferation of WB-F344 cells (**c**). The number of colonies was lower in the Bmi1-siRNA WB-F344 cells group than in the control group (**d** and **e**). Downregulation of Bmi1 expression significantly impaired the invasion of WB-F344 cells (**f**). The number of WB-F344 cells that migrated through the filter was markedly lower in the Bmi1-siRNA group than in the control group (**g**). Downregulation of Bmi1 reduced number of WB-F344 cells in the S phase of cell cycle (**h**). *P<0.05, **P<0.01.

We performed quantitative real-time polymerase chain reaction (qRT–PCR) and western blot to analyze the expression levels of Bmi1 in Bmi1-small interfering RNA (siRNA) HPCs. As shown in Figures 1a and b, Bmi1 was effectively and functionally suppressed by Bmi1-siRNA in the evaluated HPCs. To verify that Bmi1 was functionally silenced by the siRNA, we utilized western blot to analyze the expression levels of histone 2A lysine 119 ubiquitination (H2AK119ub), which is catalyzed by the Bmi1 containing polycomb-repressive complex 1. The results indicated that Bmi1-siRNA treatment led to decreased levels of H2AK119ub in HPCs (Figure 1b). We further investigated whether downregulated Bmi1 expression affected the biological activities of HPCs. A CCK-8 (cell counting kit 8) assay revealed that the decrease in Bmi1 expression significantly inhibited the proliferation of HPCs (Figure 1c). A colony-formation assay revealed that fewer colonies occurred in the Bmi1-siRNA-treated HPCs compared with the control group (Figures 1d and e). Cell invasion assay results showed that markedly fewer Bmi1-siRNA-treated HPCs migrated through a transwell filter than control group cells (Figures 1f and g). In addition, a fluorescence-activated cell sorting analysis assay revealed that Bmi1 suppression decreased the number of HPCs in S phase (Figure 1h). Collectively, these data indicate that downregulation of Bmi1 inhibits the proliferation and invasion of HPCs. *P<0.05, **P<0.01.

### Stable forced expression of Bmi1 enhances the proliferation, colony formation, invasion and cell cycle progression of HPCs *in vitro*

**Figure 2:**
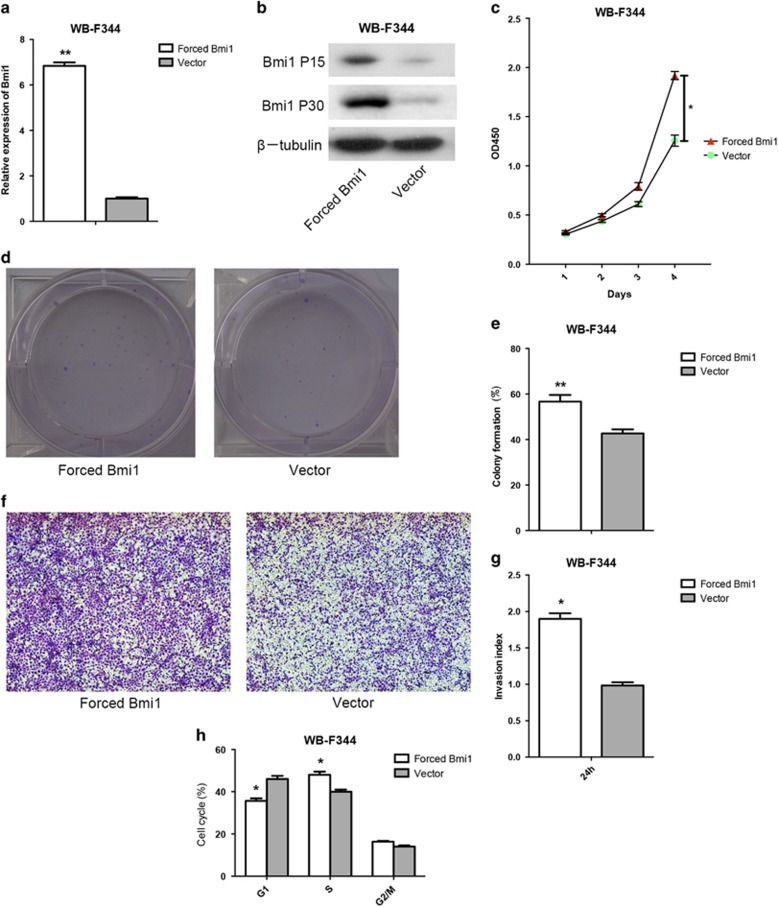
Stable forced Bmi1 expression enhanced the proliferation, colony formation, invasion and cell cycle progression of WB-F344 cells *in vitro*. The qRT–PCR analysis revealed that the mRNA expression levels of Bmi1 were increased in forced Bmi1 expression WB-F344 cells compared with control cells (**a**). Similar results were obtained through western blot analysis (passages 15 and 30) (**b**). Upregulation of Bmi1 enhanced the proliferation (**c**), colony-formation ability (**d**, **e**) and invasion (**f**) of WB-F344 cells. The number of WB-F344 cells that migrated through the filter was markedly higher in the forced Bmi1 expression group than in the control group (**g**). Forced expression of Bmi1 increased the number of WB-F344 cells in the S phase of cell cycle (**h**). *P<0.05, **P<0.01.

To investigate the effects of Bmi1 on HPCs, stable cell lines with forced Bmi1 expression were established. qRT–PCR and western blot analysis revealed that Bmi1 expression was increased in forced Bmi1 expression HPCs compared with control cells (Figures 2a and b). We further investigated whether forced Bmi1 expression was associated with HPCs biological activity. We found that the forced expression of Bmi1 increased the proliferation (Figure 2c) and colony-formation ability (Figures 2d and e) of HPCs compared with the control cells. Furthermore, the number of HPCs that migrated through the filter was greater in the forced Bmi1 expression group than the control group (Figures 2f and g). In addition, fluorescence-activated cell sorting analysis revealed that the forced expression of Bmi1 increased the number of HPCs in S phase compared with the control cells (Figure 2h). Taken together, these data suggest that forced Bmi1 expression enhances the proliferation and invasion of HPCs *in vitro*. *P<0.05, **P<0.01.

### Forced Bmi1 expression HPCs possess CSCs-like properties

**Figure 3:**
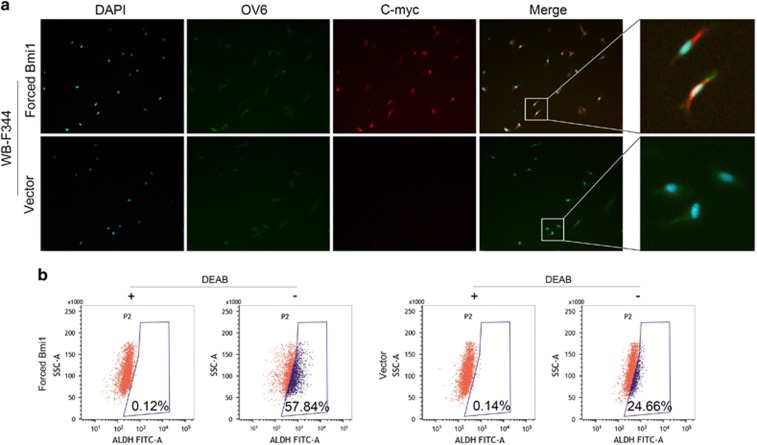
Forced Bmi1 expression WB-F344 cells possess properties of CSCs. Immunofluorescence staining revealed that the expression of c-Myc was much higher in forced Bmi1 expression WB-F344 cells than in controls, and the presence of OV6 was verified in forced Bmi1 expression WB-F344 cells (**a**). The activity of ALDH in forced Bmi1 expression WB-F344 cells and the control cells were measured with an AldeflourTM assay with and without DEAB (the specific inhibitor of ALDH) (**b**). **P<0.01.

**Figure 4:**
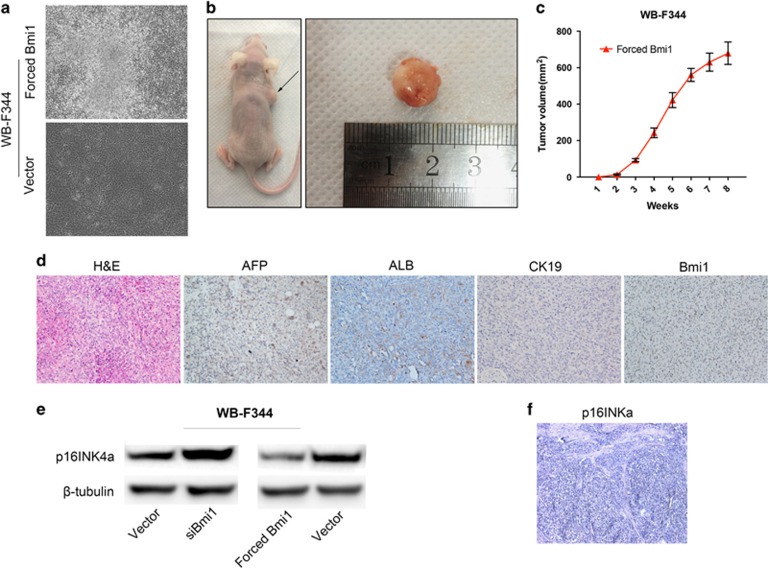
Bmi1 drives malignant transformation of WB-F344 cells *in vivo*. Forced Bmi1 expression WB-F344 cells showed a piled-up appearance in culture (**a**). Forced Bmi1 expression WB-F344 cells generated tumors on the right flanks of every tested nude mouse (**b**, black arrow), whereas no tumors were observed on the left flanks injected with negative control cells. The tumor volume of the recipient mice is presented (**c**). The histological features of the tumors corresponded to poorly differentiated HCC (**d**). The expression levels of Bmi1, AFP and albumin were much higher in subcutaneous tumors, whereas CK19 was not expressed in subcutaneous tumors (**d**). The expression level of p16Ink4a in Bmi1-siRNA WB-F344 cells and forced Bmi1 expression WB-F344 cells were analyzed by western blot (**e**). The expression of p16Ink4a in subcutaneous tumors was detected by immunohistochemistry (**f**). Magnification: × 200. *P<0.05, **P<0.01.

CSCs with stem/progenitor cell characteristics possess excessive self-renewal capability. Immunofluorescence staining revealed that the expression of the liver stem cell markers OV6 and the oncogene c-Myc was increased in the forced Bmi1 expression HPCs (Figure 3a). The aldehyde dehydrogenase (ALDH) assay, a method for prospectively isolating normal and malignant stem cells based on high intracellular expression of ALDH using the Aldefluor assay, was performed to further determine the characteristics of forced Bmi1 expression HPCs. The ALDH assay revealed that the average percentage of ALDH-positive cells was 55.84±3.52% in forced Bmi1 expression HPCs and 24.32±2.46% in the control cells (*P*<0.01); representative fluorescence-activated cell sorting images are shown in Figure 3b. In addition, forced Bmi1 expression HPCs showed a piled-up appearance *in vitro* (Figure 4a). Based on these findings, we speculated that Bmi1 facilitated hepatic CSCs-like phenotypes in HPCs and that stable the forced expression of Bmi1 drives the malignant transformation of HPCs *in vitro*.

### HPCs forced expression of Bmi1 developed HCC *in vivo*

Because our *in vitro* studies showed that Bmi1 upregulation significantly enhanced HPCs proliferation, colony formation and invasion, we utilized a xenograft-formation assay to investigate whether Bmi1 mediates tumorigenicity. As shown in Figure 4b, forced Bmi1 expression HPCs were able to generate tumors on the right flank of every nude mouse tested (black arrow), whereas no tumors were found in the left flanks, which were injected with control cells. Histological analysis (hematoxylin and eosin staining) of the subcutaneous tumors showed a disorganized arrangement of tumor cells, the presence of hyperchromatic nuclei and a high nuclear-cytoplasmic ratio. These histologic features were representative of poorly differentiated HCC (Figure 4d). To further evaluate the characteristics of the subcutaneous tumors, the expression levels of Bmi1, AFP, albumin and CK19 were analyzed through immunohistochemistry. Interestingly, the tumor cells were found to express Bmi1, AFP and albumin, but not CK19 (Figure 4d). These findings confirmed that forced Bmi1 expression resulted in malignant transformation of HPCs and promoted the initiation of HCC. *P<0.05, **P<0.01.

### Forced Bmi1 expression downregulates p16Ink4a expression in HPCs

Next, we investigated the potential mechanism underlying the effects of Bmi1 on the malignant transformation of HPCs. Bmi1 has been reported to at least partially regulate cell cycle progression through transcriptional regulation of the p16Ink4a (cyclin-dependent kinase inhibitor 2A, CDKN2A). Therefore, we examined whether Bmi1 affected the expression of p16Ink4a in HPCs. Western bolt analysis revealed that the suppression of Bmi1 in HPCs enhanced the expression of p16Ink4a (Figure 4e, left). Moreover, forced expression of Bmi1 decreased p16Ink4a expression in HPCs compared with the control cells (Figure 4e, right). Most importantly, the expression of p16Ink4a could not been detected in subcutaneous tumors by immunohistochemistry (Figure 4f). These findings indicated that Bmi1 promoted the malignant transformation of HPCs partially through its effects on p16Ink4a expression HPCs.

## Discussion

Cancer typically has a unicellular origin, although there is functional heterogeneity in tumor cells in a wide variety of cancers. Classically, two general models of the carcinogenic process have been proposed.^25^ The first is a stochastic model, which suggests that a small number of cells acquire proliferative potential via stochastic events, and these cells are then responsible for tumor formation. Alternatively, there is a hierarchical model, which postulates that a small subset of cells forms a hierarchical organization that contains varied downstream descendants. These descendants proliferate extensively and initiate tumorigenesis at a high frequency. This subset of cells is generally referred to as either tumor-initiating cells or CSCs, and they possess stem cell-like properties and form hierarchical structures containing various progenies in a manner that is similar to normal stem cells. In addition, these cells govern chemoresistance, recurrence and metastasis in various types of tumors.^26^

HPCs are involved in a broad range of human liver diseases and can bi-directionally differentiate into hepatocytes and cholangiocytes. Furthermore, these cells cannot form tumors when injected into nude mice.^27, 28, 29^ Bmi1 has been previously reported to be a crucial regulator of stem cell self-renewal and is upregulated in various types of cancers, including breast, pancreas and colon cancers as well as HCC.^6, 13, 14, 23^ However, the role of Bmi1 on HPCs has not yet been well documented. In the current study, we found that depletion of Bmi1 resulted in decreased proliferation, colony formation and invasion in HPCs *in vitro*. In contrast, the proliferation, colony formation and invasion capacities of the cells were markedly enhanced after the forced expression of Bmi1. Taken together, these findings indicate that Bmi1 has a significant role in HPCs proliferation and invasion.

Bmi1 is also critical for the malignant transformation of stem cells.^6, 7, 16^ ALDH activity, which is a marker of CSCs, has been shown to be elevated in several tumor types, including brain, breast, liver, colon, pancreas and lung cancers.^30^ Overall, isolation of ALDH-positive cells from these tumors results in enrichment of tumor-initiating cells.^31^ In the present study, the ALDH assay revealed a significant increase in the number of ALDH-positive cells following forced Bmi1 expression in HPCs. Accordingly, the oncogene marker c-Myc was enhanced by Bmi1 in HPCs. In addition, forced Bmi1 expression HPCs showed a piled-up appearance. Based on these findings, we speculated that Bmi1 upregulation leads HPCs to adopt a CSCs-like phenotype.

It has been reported that upregulation of Bmi1 in hepatic fetal stem cells can result in HCC.^32^ Bmi1 was shown to be required for liver tumor development in a chemically induced hepatocarcinogenesis mouse model.^33^ However, it is well known that HCC mainly occurs in adulthood. Importantly, in the present study, implantation of forced Bmi1 expression HPCs into nude mice produced tumors that exhibited the histologic features of poorly differentiated HCC. These findings provide the first evidence that the forced expression of Bmi1 results in the malignant transformation of HPCs, suggesting the importance of Bmi1 during hepatocarcinogenesis.

The molecular mechanisms underlying Bmi1's role in cancer progression are not completely understood. Bmi1 at least partially regulates cell cycle progression through the transcriptional regulation of the p16Ink4a.^34, 35^ Silencing of p16Ink4a expression has been implicated as a key event in HCC progression^36, 37^ and it has been reported that Bmi1 collaborates with c-Myc in lymphoma tumorigenesis via p16Ink4a.^35^ In the present study, we found that forced expression of Bmi1 in HPCs led to the downregulation of p16Ink4a. Moreover, the expression of c-Myc was upregulated by Bmi1 in HPCs. Based on these findings, we hypothesize that Bmi1 promotes the malignant transformation of HPCs, perhaps partly through its alteration of p16Ink4a expression. However, the exact mechanism underlying the role Bmi1 in the malignant transformation of HPCs requires further study.

In conclusion, our findings indicate that forced expression of Bmi1 promotes malignant transformation of HPCs. In this regard, Bmi1 might be a potential molecular target for the treatment of HCC.

## Materials and methods

### Cell lines and cell culture

Two HPC cell lines were used in this study: WB-F344 was obtained from the Cell Bank of the Chinese Academy of Sciences (Shanghai, China) and the (OC3) hepatic oval cell line 3, which was established from a Sprague-Dawley rat model as previously described.^29, 38^ The morphological and biological properties of these cell lines have been previously characterized as mainly resembling those of HPCs.^27, 39^

The HPCs were cultured in DMEM/F12 (Invitrogen Co., Carlsbad, CA, USA) supplemented with 10% heat-inactivated fetal bovine serum (Invitrogen), as recommended by the supplier. All of the cultures were maintained in a humidified atmosphere containing 5% CO_2_ at 37 °C. The HPCs used in this study were grown from low passages to passage 25 *in vitro*. Only the cells in logarithmic growth phase were used throughout the research.

### Small interfering RNA transfection

A validated siRNA targeting Bmi1 and the negative control (listed in Table 1) were purchased from GenePharma (Shanghai, China). All of the siRNA sequences were subjected to Basic Local Alignment Search Tool analysis to confirm the absence of homology to any additional known coding sequences in the rat genome. Transfection was performed as described previously.^11^ Knockdown of Bmi1 was confirmed through qRT–PCR and western blot analyses.

### Retroviral transfection and stable cell line generation

Full-length rat Bmi1 complementary DNA was PCR amplified from total rat embryo complementary DNA and hemagglutinin-tagged, followed by insertion into the pMSCV vector immediately upstream of the internal ribosome entry site. The Bmi1 fragment was then isolated through NotI digestion and inserted into a Bmi1 retroviral vector (pMSCV/Bmi1). The HPCs were infected at 10 population doublings with pMSCV/Bmi1 or the pMSCV control. After puromycin selection, the forced expression of Bmi1 was confirmed through qRT–PCR and western blot analyses from passages 5 to 30. The forced Bmi1 expression HPCs used in this study was grown from passages 10 to 25 *in vitro*.

### Quantitative RT–PCR analysis

Total RNA was isolated using the RNAiso Plus reagent according to the manufacturer's protocol (TaKaRa, Tokyo, Japan). The primer sets employed for PCR amplification are shown in Table 2. As a control, the levels of glyceraldehyde phosphate dehydrogenase expression were also analyzed. qRT–PCR was performed as described previously.^11^

### Western blot analysis

Western blot analysis was performed as described previously.^11^ In brief, cell pellets were washed three times with ice-cold phosphate-buffered saline (PBS) and lysed with RIPA buffer (Beyotime, Guangzhou, China). The total protein concentration was determined using a bicinchoninic acid assay kit (Beyotime). Samples were denatured in 5 × sodium dodecyl sulfate-sample buffer at 95 °C for 5 min. Equal amounts of total proteins were separated through 12% sodium dodecyl sulfate polyacrylamide gel electrophoresis and then transferred to polyvinylidene difluoride membranes. The membranes were then blocked with 5% dried skimmed milk in PBS with Tween 20 at room temperature for 1 h. After blocking, the membranes were incubated with corresponding antibodies recognizing Bmi1 (1:300), β-tubulin (1:1000) and p16Ink4a (1:500) overnight at 4 °C. After washing three times with PBS with Tween 20, the membranes were incubated with the appropriate horseradish peroxidase-conjugated secondary antibodies and washed three times with PBS with Tween 20 again. The proteins were detected using ECL plus reagents (Beyotime). Details regarding the primary and secondary antibodies are provided in Supplementary Table 1. The results of western blot were quantified by ImageJ (Supplementary Figure 1).

### Cell proliferation assay

HPCs (5 × 10^3^/well) were cultured in 96-well tissue culture plates until they reached 50% confluence, then transfected with a final concentration of 100 nM. The proliferation abilities of HPCs were determined by CCK-8 assays (Dojindo Molecular Technologies, Gaithersburg, MA, USA). In brief, 10 μl of water-solubleformazan dye was added to each well and incubated for 2 h. The absorbance at 450 nm was measured by an enzyme linked immunosorbent assay plate reader. The absorbance of the negative control (OD) was considered to be 0%.

### Flow cytometry

The cell cycle was analyzed using flow cytometry with propidium iodide (Sigma, California, CA, USA) staining. In each group, cells were harvested and washed with PBS and fixed overnight in ice-cold 70% ethanol. The cells were then washed twice with PBS and treated with 1 mg/l RNaseA (TaKaRa) for 15 min. Finally, the cells were stained with 50 mg/l propidium iodide in the dark for 1 h. Cell cycle analysis was subsequently performed with a fluorescence-activated cell sorter (BD Biosciences, Franklin Lakes, NJ, USA), and propidium iodide fluorescence was measured at 488 nm. Each group was analyzed in triplicate, and at least 10 000 cells were analyzed in each experiment.

### ALDH detection assay

ALDH staining was performed with the ALDEFLUORTM kit (Stem Cell Technologies, Vancouver, BC, Canada) according to the manufacturer's instructions. In brief, cells were suspended at 1 × 10^6^ cells/ml in Aldefluor assay buffer containing ALDH substrate (BAAA, BODIPY aminoacetaldehyde, 1 mmol/l), with or without the specific ALDH inhibitor diethylaminobenzaldehyde (1 mmol/l) for 30 min. Diethylaminobenzaldehyde serves as internal negative control for each individual experiment, and allows to distinguish between ALDH-bright (ALDH positive) cells and cells with low ALDH activity (ALDH negative). Analysis and sorting were conducted on a fluorescence-activated cell sorting: Aldefluor was excited at 488 nm and fluorescence emission was detected at 530/30. Dead cells were excluded by gating on forward and side scatter and eliminating the propidium iodide-positive population. The data were analyzed by Cell Quest Pro and FlowJo (Ashland, OR, USA).

### Colony-formation assay

A total of 200 HPCs every well were seeded into a fresh six-well plate and incubated in DMEM/F12 containing 10% fetal bovine serum. After 7 days, the cells were rinsed with PBS twice, fixed with 10% formaldehyde, and stained with 0.1% crystal violet in 10% ethanol. Microscopic colonies composed of >50 cells were counted in each well.

### Cell invasion assay

The invasive activity of HPCs was estimated using transwells (6.5 mm in diameter, polycarbonate membrane, 8 μm pore size) coated with extracellular matrix gel obtained from Corning Inc. (Corning, NY, USA). After 24 h of transfection, an aliquot of 1 × 10^5^ cells was placed in the upper chamber with 0.1 ml serum-free medium, whereas the lower chamber (24-well plate) was loaded with 0.5 ml of medium containing 10% fetal bovine serum. After 24 h of incubation, the cells were fixed with 4% paraformaldehyde and then counterstained with 0.1% crystal violet. The cells that had migrated into the lower chamber were observed and counted under a light microscope. Then the number of migratory cells was calculated.

### *In vivo* tumorigenicity assays

All of the animal experimentation described in this study was performed in accordance with protocols approved by the Institutional Animal Care and Use Committee at Sun Yat-sen University. A total of 5 × 10^6^ forced Bmi1 expression HPCs and control cells(WB-F344 vector cells or OC3 vector cells) were suspended in 100 μl of PBS and then injected subcutaneously into eight female nude mice (Balb/c nu/nu) (3–4-week old). Tumor volumes were monitored every 7 days by measuring the tumor length and width with calipers and were calculated using the formula (width^2^) × length/2. The mice were killed 8 weeks after the injections, and the tumors were isolated and measured. Paraffin-embedded sections of murine tumors were stained with hematoxylin and eosin.

### Immunohistochemistry and immunofluorescence staining

Tumors that formed in nude mice were fixed in formalin and embedded in paraffin. Sections of paraffin-embedded tissue were stained with Bmi1, AFP, Albumin, CK19 and p16Ink4a by immunohistochemistry as described before.^18^ The expression of liver stem cell markers OV6 and oncogene c-Myc were also detected by immunofluorescence staining in forced Bmi1 expression HPCs as described before.^11^ The primary antibodies and secondary antibodies listed in Supplementary Table 1.

### Statistics

All statistical analysis was performed using SPSS for Windows (version 17.0, SPSS, Chicago, IL, USA). All experiments for cell cultures were carried out independently at least three times and in triplicate each time. All data were expressed as mean±s.d. unless otherwise indicated. In the *in vitro* data using Student's *t*-test, and in the *in vivo* data using the Mann–Whitney *U*-test. In all cases, *P*-values of <0.05 and 0.01 were considered statistically significant.

## Figures and Tables

**Table 1:** Sequences of siRNAs for Bmi1

*Gene*	*Primer*	*Sequence*
Bmi1-siRNA	Forward	5′-GCU UAGAGAUGCUAAUACATT-3′
	Reverse	5′-UGUAUUAGCAUCUCUAAGCTT-3′
Bmi1-negative control	Forward	5′-UUGUCCGAACGUGUCACGUTT-3′
	Reverse	5′-ACGUGACACGUUCGG AGA ATT-3′

**Table 2:** Primers for Bmi1 and reference genes

*Gene*	*Primer*	*Sequence*
Bmi1	Forward	5′-AGCAGCAATGACTGTGATGC-3′
	Reverse	5′-CAGTCTCAGGTATCAACCAG-3′
GAPDH	Forward	5′-GCACCGTCAAGGCTGAGAAC-3′
	Reverse	5′-TGGTGAAGACGCCAGTGGA-3′

## Supplementary Material

Supplementary Infomation
