# Supplementary material for: Dysregulation of Bmi1 promotes malignant transformation of hepatic progenitor cells
Source: Oncogenesis. 2016 Feb 29;5(2):e203–. doi: 10.1038/oncsis.2016.6 (PMC5154353; doi:10.1038/oncsis.2016.6)
Supplement: Supplementary Infomation [file oncsis20166x1.doc]

**Supplementary Information:**

**Supplementary figure 1** The results of western blot were quantified by ImageJ.

The protein levels of Bmi1 in HPCs were quantified by ImageJ (A-C). The protein levels of H2AK119µb in Bmi1-siRNA HPCs were quantified by ImageJ (D). The protein levels of p16Ink4a in HPCs were quantified by ImageJ (E-F). *P<0.05, **P<0.01.

**Supplementary figure 2** **Transfection of Bmi1-siRNA represses proliferation, colony formation and cell cycle progression of OC3 cells in vitro**

The qRT-PCR analysis revealed that the mRNA expression levels of Bmi1 in Bmi1-siRNA OC3 cells was significantly downregulated (A). Similar results appeared in western blot analysis (B). The expression of H2AK119ub was downregulated in OC3 by Bmi1-siRNA (B). Down-regulation of Bmi1 expression significantly inhibits the proliferation (C) and colony formation (D) of OC3 cells. The number of colony was lower in Bmi1-siRNA OC3 cells group than the control group (E). Downregulation of Bmi1 expression significantly impaired the invasion of OC3 cells (F). The number of OC3 cells that migrated through the filter was markedly lower in the Bmi1-siRNA group than in the control group (G). Down-regulation of Bmi1 reduced number of OC3 cells in the S phase of cell cycle (H). *P<0.05, **P<0.01.

**Supplementary figure 3.** **Stable forced Bmi1 expression enhanced proliferation, colony formation and cell cycle progression of OC3 cells in vitro**

The qRT-PCR analysis and western blot analysis (passages15 and 30) revealed that the expression level of Bmi1 was increased in forced Bmi1 expression OC3 cells compared with control cells (A-B). Up-regulation of Bmi1 enhanced proliferation (C), colony formation ability (D) and invasion (F) in forced Bmi1 expression OC3 cells compared with control cells. The number of colony (E) and the number of OC3 cells that migrating through the filter (G) was markedly higher in forced Bmi1 expression group than the control group. Forced expression of Bmi1 increased number of OC3 cells in the S phase of cell cycle (H). *P<0.05, **P<0.01.

**Supplementary figure 4. Forced Bmi1** **expression OC3 possess properties of CSCs**

Immunofluorescence staining revealed that the expression of c-Myc was much higher in forced Bmi1 expression OC3 cells than in controls (A). The activity of ALDH in forced expression OC3 cells and the control cells were measured with an AldeflourTM assay with and without DEAB (the speciﬁc inhibitor of ALDH) (B). **P<0.01.

**Supplementary figure 5.Bmi1 drives malignant transformation of OC3 cells in vivo.**

Forced Bmi1 expression OC3 cells generated tumors on the right flanks of every tested nude mouse (A, black arrow), whereas no tumors were observed on the left flanks injected with negative control cells. The tumor volume of the recipient mice is presented (B). The histological features of the tumors corresponded to poorly differentiated HCC (C). The expression levels of Bmi1, AFP and albumin were much higher in subcutaneous tumors, whereas CK19 was not expressed in tumors (D). The expression level of p16Ink4a in Bmi1-siRNA OC3 cells and forced Bmi1 expression OC3 cells were analyzed by western blot (E). The expression of p16Ink4a in subcutaneous tumors was detected by immunohistochemistry (F). Magnification:×200. **P<0.01.
