# Supplementary figures and images for: Dysregulation of Bmi1 promotes malignant transformation of hepatic progenitor cells
Source: Oncogenesis. 2016 Feb 29;5(2):e203–. doi: 10.1038/oncsis.2016.6 (PMC5154353; doi:10.1038/oncsis.2016.6)

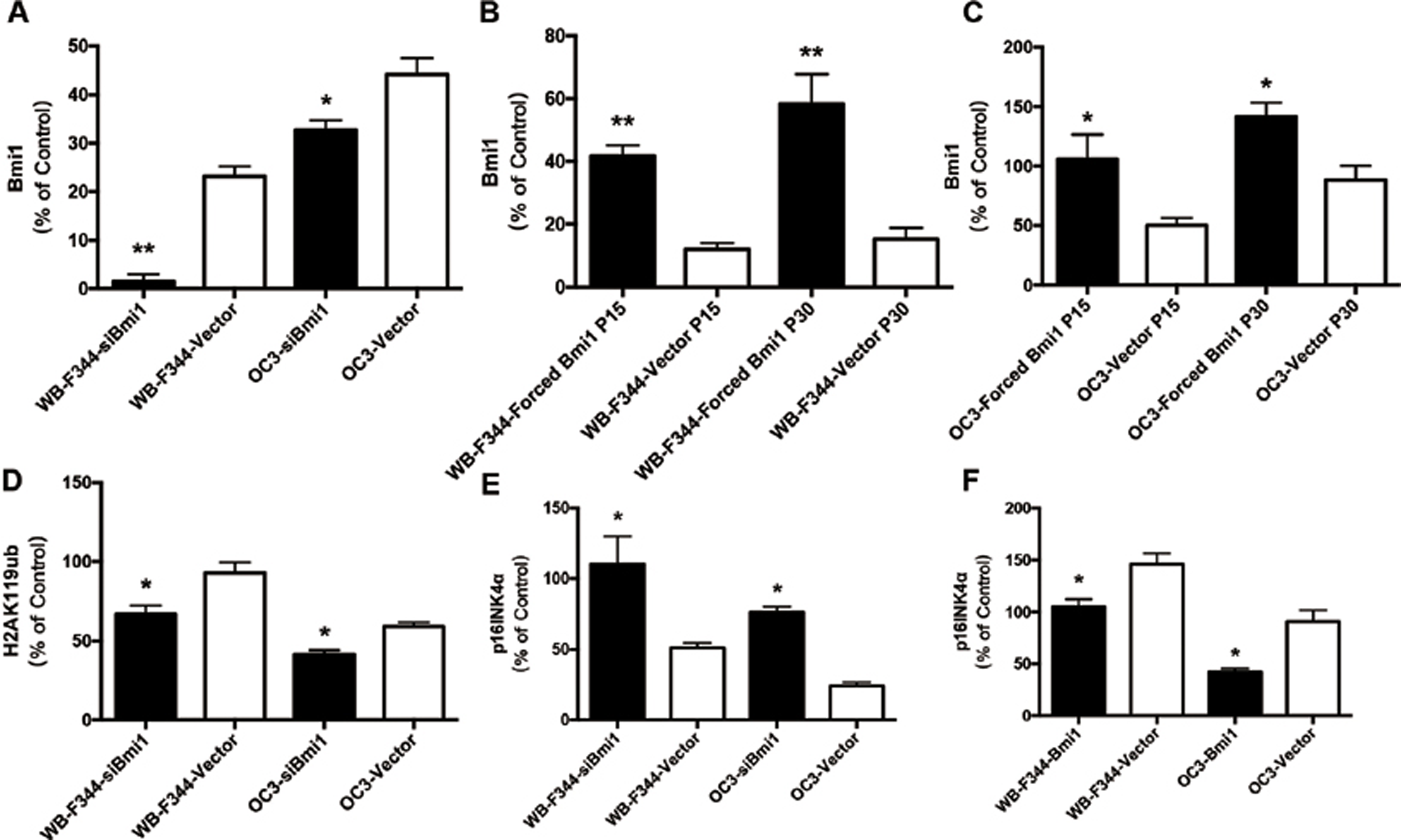

Supplement: Supplementary Figure 1 [file oncsis20166x2.tif]

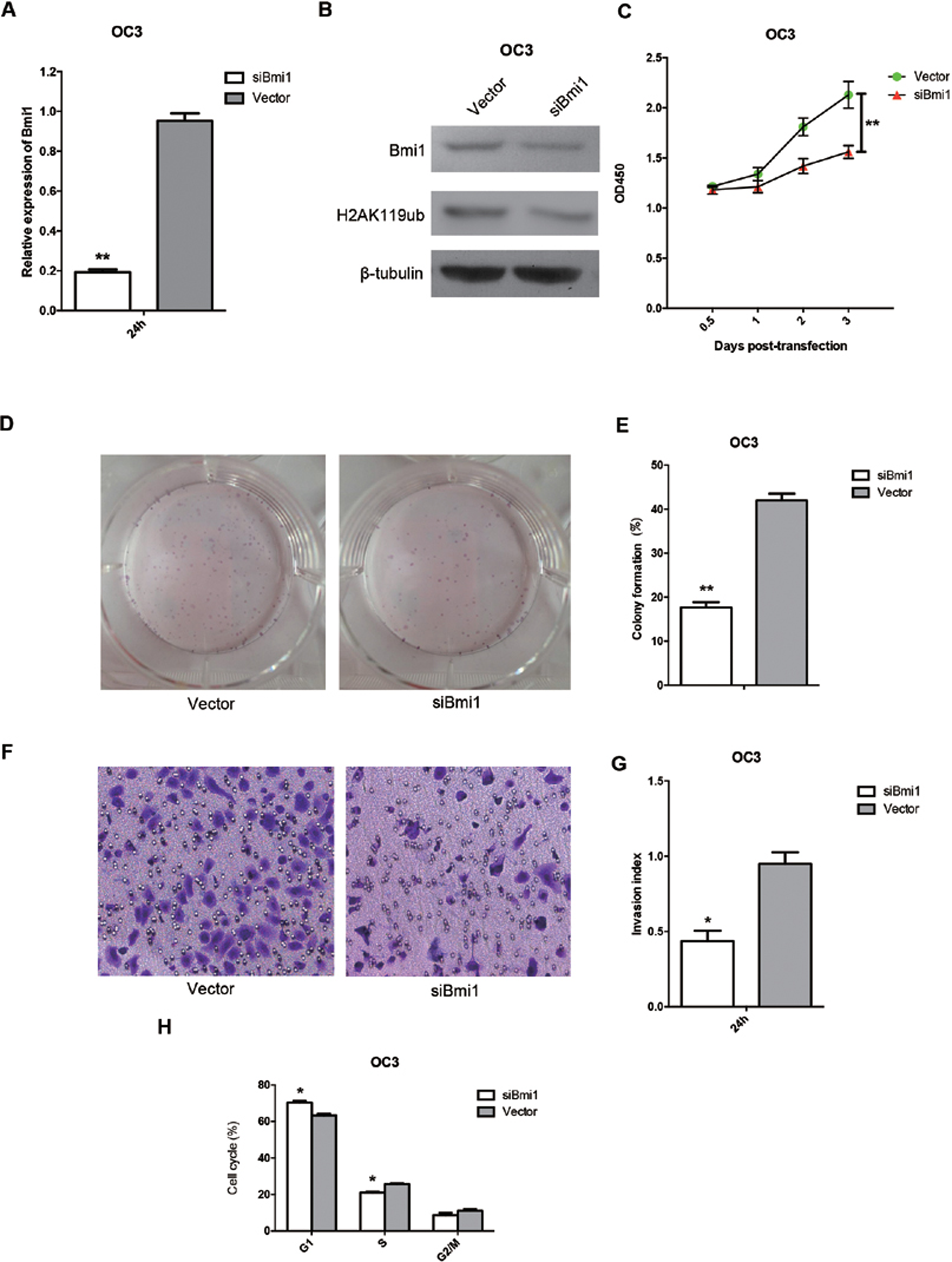

Supplement: Supplementary Figure 2 [file oncsis20166x3.tif]

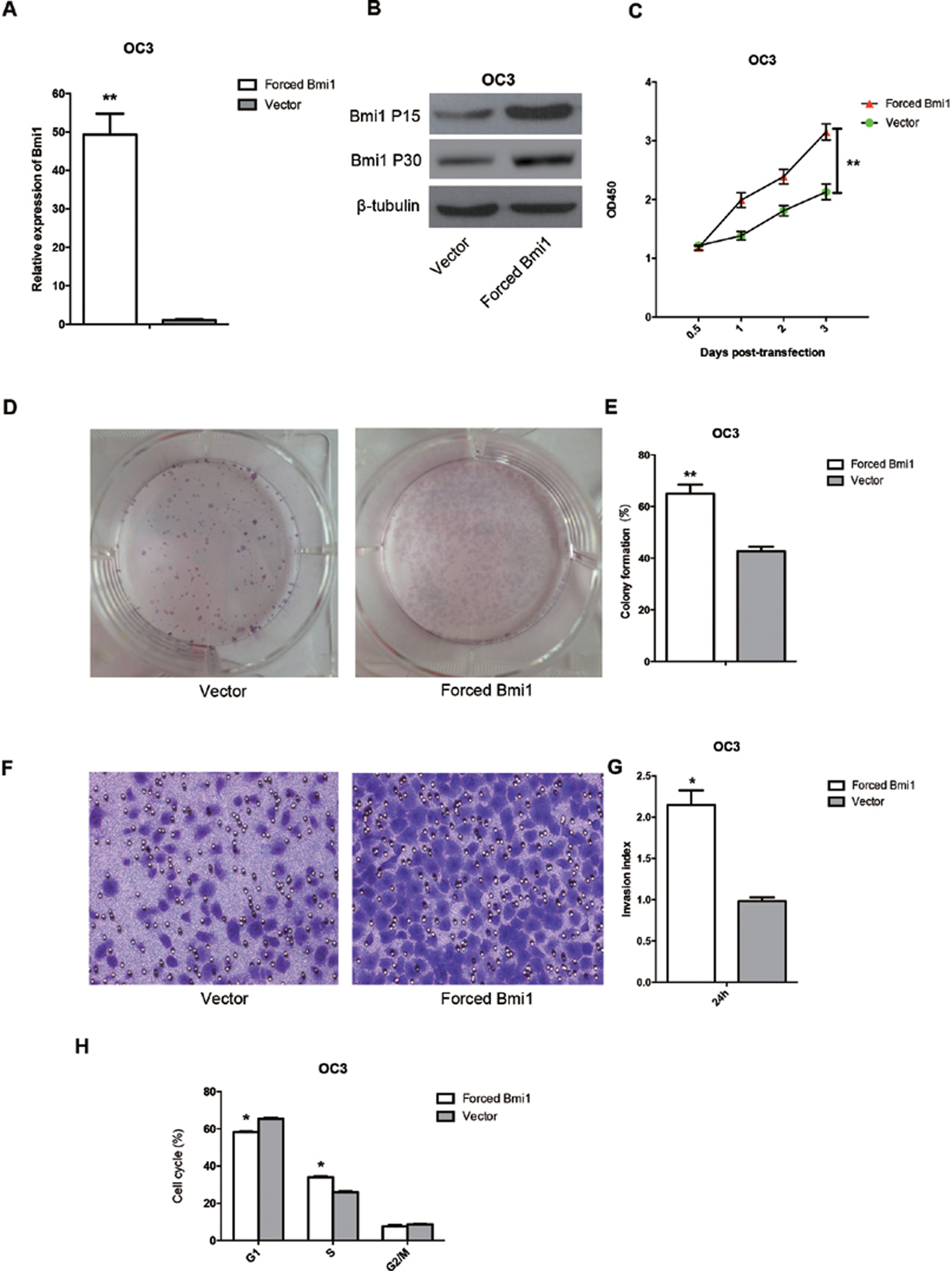

Supplement: Supplementary Figure 3 [file oncsis20166x4.tif]
