# Supplementary material for: Dysregulation of Bmi1 promotes malignant transformation of hepatic progenitor cells
Source: Oncogenesis. 2016 Feb 29;5(2):e203–. doi: 10.1038/oncsis.2016.6 (PMC5154353; doi:10.1038/oncsis.2016.6)
Supplement: Supplementary Table 1 [file oncsis20166x7.doc]

**Supplementary Table 1** The information of antibodies used in this study

| Antibodies | Company | Product Number | Dilution |
| --- | --- | --- | --- |
| Bmi1 | Santa Cruz | SC-10745 | 1:300 (WB) 1:200 (IHC) |
| c-Myc | Santa Cruz | SC-7870 | 1:500 (IF) |
| β-tubulin | Cell Signaling Technology | #2125 | 1:1000 (WB) |
| HRP-linked anti-rabbit IgG | Cell Signaling Technology | #7074 | 1:2000 (WB) |
| HRP-linked anti-mouse IgG | Cell Signaling Technology | #7076 | 1:2000 (WB) |
| OV6 | R&D systems | MAB-2020 | 1:500 (IF) |
| AFP | Santa Cruz | SC-8108 | 1:50 (IHC) |
| Albumin | Zhongshan | ZS-1902 | 1:200(IHC) |
| CK19  p16Ink4a | Zhongshan  Abcam | ZS-2507  ab117443 | 1:200(IHC)  1:500 (WB) 1:100 (IHC) |
| Cy3-AffiniPure Donkey anti-goat IgG | Jackson ImmunoResearch | 705-165-003 | 1:400 (IF) |
| Alexa Fluor 488-AffiniPureDonkey anti-mouse IgG | Jackson ImmunoResearch | 715-545-151 | 1:400 (IF) |
